# Supplementary material for: Heat adaptation of phage T7 under an extended genetic code
Source: Virus Evol. 2021 Dec 1;7(2):veab100. doi: 10.1093/ve/veab100 (PMC8923235; doi:10.1093/ve/veab100)
Supplement: veab100_Supp [file veab100_supp.zip › Sequencing_Analysis.zip!/Sequencing_Analysis/A1/summary.html]

BRESEQ :: Summary Statistics


|  |  |
| --- | --- |
|  | ***breseq***  version 0.30.1    mutation predictions \| marginal predictions \| summary statistics \| genome diff \| command line log |

## Read File Information

|  | read file | reads | bases | passed filters | average | longest | mapped |
| --- | --- | --- | --- | --- | --- | --- | --- |
| errors | A1\_52\_S13\_L001\_R1\_001 | 510,741 | 153,733,041 | 100.0% | 301.0 bases | 301 bases | 78.5% |
|  | **total** | **510,741** | **153,733,041** | 100.0% | 301.0 bases | **301** bases | 78.5% |

## Reference Sequence Information

|  |  | seq id | length | fit mean | fit dispersion | % mapped reads | description |
| --- | --- | --- | --- | --- | --- | --- | --- |
| coverage | distribution | NC\_001604.1 | 39,937 | 3071.8 | 62.9 | 100.0% | na |
|  |  | **total** | **39,937** |  |  | 100.0% |  |

**fit dispersion** is the ratio of the variance to the mean for the negative binomial fit. It is =1 for Poisson and >1 for over-dispersed data.

## New Junction Evidence

### Junction Candidates Tested

| option | limit | actual |
| --- | --- | --- |
| Number of alignment pairs examined for constructing junction candidates | ≤ 100000 | 4218 |
| Coverage evenness (position-hash) score of junction candidates | ≥ 2 | ≥ 2 |
| Test this many junction candidates (n). May be smaller if not enough passed the coverage evenness threshold | 100 ≤ n ≤ 5000 | 22 |
| Total length of all junction candidates (factor times the reference genome length) | ≤ 0.1 | 0.335 |

### Junction Skew Score Calculation

| reference sequence | pr(no read start) |
| --- | --- |
| NC\_001604.1 | 0.41141 |

**pr(no read start)** is the probability that there will not be an aligned read whose first base matches a given position on a given strand.

### Final Junction Predictions

| option | value |
| --- | --- |
| Coverage evenness (position-hash) score of predicted junctions must be | ≥ 3 |
| Skew score of predicted junction (−log10 probability of unusual coverage evenness) must be | ≤ 3 |
| Number of bases that at least one read must overlap each uniquely aligned side of a predicted junction | ≥ 1 |

## Read Alignment Evidence

| option | value |
| --- | --- |
| Mode | Consensus/Mixed Base |
| Ploidy | 1 (haploid) |
| Consensus mutation E-value cutoff | 10 |
| Consensus frequency cutoff | 0.8 |
| Consensus minimum coverage each strand | OFF |
| Polymorphism E-value cutoff | 10 |
| Polymorphism frequency cutoff | 0.2 |
| Polymorphism minimum coverage each strand | OFF |
| Polymorphism bias cutoff | OFF |
| Predict indel polymorphisms | YES |
| Skip indel polymorphisms in homopolymers runs of | OFF |
| Skip base substitutions when they create a homopolymer flanked on each side by | OFF |

## Software Versions

| program | version |
| --- | --- |
| bowtie2 | 2.2.6 |
| R | 3.4.0 |

## Execution Times

| step | start | end | elapsed |
| --- | --- | --- | --- |
| Read and reference sequence file input | 12:00:45 07 Aug 2017 | 12:00:59 07 Aug 2017 | 14 seconds |
| Read alignment to reference genome | 12:00:59 07 Aug 2017 | 12:07:30 07 Aug 2017 | 6 minutes 31 seconds |
| Preprocessing alignments for candidate junction identification | 12:07:31 07 Aug 2017 | 12:07:46 07 Aug 2017 | 15 seconds |
| Preliminary analysis of coverage distribution | 12:07:46 07 Aug 2017 | 12:08:29 07 Aug 2017 | 43 seconds |
| Identifying junction candidates | 12:08:29 07 Aug 2017 | 12:08:31 07 Aug 2017 | 2 seconds |
| Re-alignment to junction candidates | 12:08:31 07 Aug 2017 | 12:10:21 07 Aug 2017 | 1 minute 50 seconds |
| Resolving alignments with junction candidates | 12:10:21 07 Aug 2017 | 12:10:54 07 Aug 2017 | 33 seconds |
| Creating BAM files | 12:10:54 07 Aug 2017 | 12:11:27 07 Aug 2017 | 33 seconds |
| Tabulating error counts | 12:11:27 07 Aug 2017 | 12:11:51 07 Aug 2017 | 24 seconds |
| Re-calibrating base error rates | 12:11:51 07 Aug 2017 | 12:11:54 07 Aug 2017 | 3 seconds |
| Examining read alignment evidence | 12:11:54 07 Aug 2017 | 12:18:00 07 Aug 2017 | 6 minutes 6 seconds |
| Polymorphism statistics | 12:18:00 07 Aug 2017 | 12:18:02 07 Aug 2017 | 2 seconds |
| Output | 12:18:02 07 Aug 2017 | 12:19:20 07 Aug 2017 | 1 minute 18 seconds |
| **Total** | | | **18 minutes 34 seconds** |
